# Understanding and tuning blue-to-near-infrared photon cutting by the Tm^3+^/Yb^3+^ couple

**DOI:** 10.1038/s41377-020-00346-z

**Published:** 2020-06-19

**Authors:** Dechao Yu, Ting Yu, Arnoldus J. van Bunningen, Qinyuan Zhang, Andries Meijerink, Freddy T. Rabouw

**Affiliations:** 1 Debye Institute for Nanomaterials Science, Utrecht University, Princetonplein 1, 3584 CC Utrecht, The Netherlands; 2 State Key Laboratory of Luminescence Materials and Devices, Institute of Optical Communication Materials, South China University of Technology, Guangzhou, 510641 China

**Keywords:** Optical materials and structures, Optical spectroscopy

## Abstract

Lanthanide-based photon-cutting phosphors absorb high-energy photons and ‘cut’ them into multiple smaller excitation quanta. These quanta are subsequently emitted, resulting in photon-conversion efficiencies exceeding unity. The photon-cutting process relies on energy transfer between optically active lanthanide ions doped in the phosphor. However, it is not always easy to determine, let alone predict, which energy-transfer mechanisms are operative in a particular phosphor. This makes the identification and design of new promising photon-cutting phosphors difficult. Here we unravel the possibility of using the Tm^3+^/Yb^3+^ lanthanide couple for photon cutting. We compare the performance of this couple in four different host materials. Cooperative energy transfer from Tm^3+^ to Yb^3+^ would enable blue-to-near-infrared conversion with 200% efficiency. However, we identify phonon-assisted cross-relaxation as the dominant Tm^3+^-to-Yb^3+^ energy-transfer mechanism in YBO_3_, YAG, and Y_2_O_3_. In NaYF_4_, in contrast, the low maximum phonon energy renders phonon-assisted cross-relaxation impossible, making the desired cooperative mechanism the dominant energy-transfer pathway. Our work demonstrates that previous claims of high photon-cutting efficiencies obtained with the Tm^3+^/Yb^3+^ couple must be interpreted with care. Nevertheless, the Tm^3+^/Yb^3+^ couple is potentially promising, but the host material—more specifically, its maximum phonon energy—has a critical effect on the energy-transfer mechanisms and thereby on the photon-cutting performance.

## Light conversion: Insights into photon cutting

Improved understanding of ‘photon cutting’, in which a high-energy photon is absorbed by a phosphor material and converted into two or more lower-energy photons, could lead to new applications in fields ranging from experimental optics to commercial light displays. Qinyuan Zhang at South China University of Technology and Freddy Rabouw at Utrecht University in the Netherlands, with colleagues, explored photon cutting in crystals doped with ions of the lanthanide elements Thulium (Tm^3+^) and Ytterbium (Yb^3+^). They identified key energy transfer mechanisms and methods for fine-tuning the process by exploring the conversion of photons of blue light into near-infrared photons, in several phosphors. The research confirms the potential of Tm^3+^/Yb^3+^ doped materials for achieving light energy conversion efficiencies above 100 percent, but suggests earlier reports of surprisingly high efficiencies should be treated with caution.

## Introduction

Lanthanide-based phosphors offer wide possibilities for colour conversion, absorbing one colour of light and emitting another^1^. The conversion process often involves energy transfer between lanthanide dopants^2,3^. Consumer applications, such as lighting and displays, usually rely on colour conversion by conventional ‘downshifting’ luminescence: the material emits one redshifted (lower-energy) photon for each photon it absorbs. The energy level structures of the lanthanides, however, offer more colour-conversion possibilities. Unconventional energy-transfer pathways between lanthanide dopants can be designed, which lead to ‘upconversion’ luminescence^4–6^ or ‘photon cutting’^7,8^. Upconversion involves merging of the energy of multiple photons by the phosphor material, i.e., it absorbs two (or more) low-energy photons and emits one higher-energy photon. Photon cutting is the inverse process (therefore also known as ‘downconversion’), whereby one higher-energy photon is absorbed and two (or more) lower-energy photons are emitted.

This work explores new strategies to achieve photon cutting by lanthanide-doped phosphors. The process was first proposed as a concept that could drastically increase the efficiency of fluorescent lighting, offering the prospect of ultraviolet-to-visible conversion efficiencies of up to 200%^7^. However, with the advances in blue light-emitting diodes over the past two decades^9^, the societal need for new fluorescent-lighting technologies has decreased. Photon cutting has been identified as a potential method to break the Shockley–Queisser limit of 33.7% in photovoltaics^10–12^. This limit otherwise sets the maximum conversion efficiency of single-junction solar cells under standard solar irradiation, determined by the optimum balance between thermalization losses and transmission losses^13^. A photon-cutting phosphor should reshape the spectrum from the sun before it enters a solar cell by converting high-energy photons into multiple lower-energy photons. Using this concept, the maximum achievable solar-cell efficiency increases to ~40%^14^.

Photon-cutting phosphors exhibiting Yb^3+^ emission have been of particular interest, because the emission at ~10,000 cm^−1^ matches the bandgap (9000 cm^−1^) of crystalline silicon solar cells. Desirable phosphors are codoped with a sensitizer ion that absorbs in the visible spectral range and transfers its energy to two Yb^3+^ ions. Phosphors doped with Tb^3+^/Yb^3+^^8,15^, Ce^3+^/Yb^3+^^16–18^, Tm^3+^/Yb^3+^^19–23^, Pr^3+^/Yb^3+^^24,25^, and other ion couples have been proposed. However, not all types of energy-transfer process between ion couples yield two (or more) excited Yb^3+^ ions per absorption event. For example, the Ce^3+^-to-Yb^3+^ energy-transfer mechanism in codoped yttrium aluminium garnet (YAG; Y_3_Al_5_O_12_) yields only a single Yb^3+^ excitation, so YAG:Ce^3+^,Yb^3+^ is not a photon cutter despite a favourable energy-level structure^16,17^. Unfortunately, the energy-transfer mechanisms are unclear for many potential photon-cutting phosphors, resulting in contradictory^19,20^ or poorly supported interpretations in the literature^12^. This complicates the identification and optimization of promising photon-cutting materials. Photon correlation measurements are a direct way to prove photon cutting^26^. Unfortunately, these measurements are difficult for Yb^3+^ emission, because single-photon detectors with high efficiencies and low dark counts are not readily available for the near-infrared region.

Here we study the potential of photon cutting with the Tm^3+^/Yb^3+^ lanthanide couple. The existing literature makes contradictory claims about the mechanism of energy transfer from the Tm^3+ 1^G_4_ level to Yb^3+^^12^, with important implications for the photon-cutting potential of the Tm^3+^/Yb^3+^ couple. A cooperative mechanism would, but a phonon-assisted cross-relaxation mechanism would not, result in blue-to-near-infrared photon cutting with the potential to increase the current output of crystalline Si solar cells^10–12^. We measure and model the dynamics of Tm^3+^-to-Yb^3+^ energy transfer in four different host materials with systematically varied doping concentration. We identify phonon-assisted cross-relaxation as the dominant energy-transfer mechanism in Tm^3+^/Yb^3+^-codoped YBO_3_, YAG, or Y_2_O_3_. In contrast, cooperative energy transfer dominates in Tm^3+^/Yb^3+^-codoped NaYF_4_. Consequently, only NaYF_4_ is a promising host material to achieve photon cutting for silicon photovoltaics with the Tm^3+^/Yb^3+^ couple. We can rationalize our results by considering the maximum phonon energies of the four host materials:^6,27^ the more phonons required for phonon-assisted cross-relaxation, the lower the rate^28^. Our work highlights the possibility of tuning the energy-transfer pathways in lanthanide-based phosphors with the appropriate choice of host material and thereby achieving photon-conversion efficiencies above 100%.

## Results

**Fig. 1:**
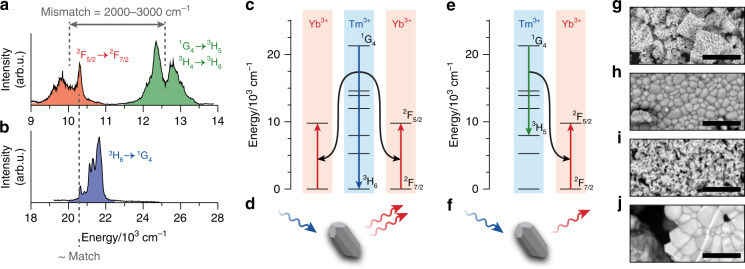
Basic properties of Tm^3+^/Yb^3+^ codoped phosphors. **a** Emission spectrum of YBO_3_ doped with 0.1% Tm^3+^ and 2% Yb^3+^ upon excitation of the Tm^3+ 1^G_4_ level at 465 nm. The red-shaded band is due to the ^2^F_5/2_ → ^2^F_7/2_ emission of Yb^3+^, and the green-shaded band is due to the overlapping ^1^G_4_ → ^3^H_5_ and ^3^H_4_ → ^3^H_6_ emissions of Tm^3+^^31^. **b** Corresponding excitation spectrum, measured by scanning through the Tm^3+ 3^H_6_ → ^1^G_4_ absorption transition and recording the intensity of the ^1^G_4_ → ^3^F_4_ emission at 653 nm (15,300 cm^−1^). **c** Cooperative energy transfer involves the distribution of the excited-state energy in the Tm^3+ 1^G_4_ level over two nearby Yb^3+^ ions. **d** This can eventually yield two near-infrared photons of 1000 nm emitted by Yb^3+^ per blue photon absorption event. **e** A Tm^3+^ ion in the ^1^G_4_ level can alternatively transfer part of its energy to a single nearby Yb^3+^ ion through phonon-assisted cross-relaxation. **f** This produces at most one near-infrared photon of 1000 nm. **g**–**j** Scanning electron micrographs of the four host materials in order of decreasing highest phonon energy: **g** YBO_3_, **h** YAG, **i** Y_2_O_3_, and **j** NaYF_4_. The scale bars represent 5 μm

To investigate the Tm^3+^-to-Yb^3+^ energy-transfer mechanism in Tm^3+^/Yb^3+^-codoped YBO_3_, YAG, Y_2_O_3_, and NaYF_4_, we recorded luminescence spectra (emission and excitation) and luminescence decay curves. As an example, Fig. 1a shows the emission spectrum of microcrystalline YBO_3_ doped with 0.1% Tm^3+^ and 2% Yb^3+^ upon excitation in the blue region (at 465 nm; 21,500 cm^−1^). This spectrum shows similar features to those previously measured on Tm^3+^/Yb^3+^-codoped borates and other host materials^29–31^. The emission line centred at 10,000 cm^−^^1^ (shaded red) is due to the ^2^F_5/2_ → ^2^F_7/2_ transition of Yb^3+^, and that centred at 12,500 cm^−^^1^ (shaded green) is due to the energetically overlapping ^1^G_4_ → ^3^H_5_ and ^3^H_4_ → ^3^H_6_ transitions of Tm^3+^. The appearance of Yb^3+^-based emission following excitation of the Tm^3+ 1^G_4_ level (see Fig. 1b for an excitation spectrum) evidences the occurrence of energy transfer from Tm^3+^ to Yb^3+^.

Many previous studies on Tm^3+^/Yb^3+^-codoped materials have concluded that Tm^3+^-to-Yb^3+^ energy transfer follows the cooperative mechanism (Fig. 1c)^19,21–23^: an excited Tm^3+^ dopant in the ^1^G_4_ level transfers its energy in a single step to two nearby Yb^3+^ dopants. This process brings the Tm^3+^ donor back to its ^3^H_6_ ground state and excites both Yb^3+^ acceptor ions to their ^2^F_5/2_ excited state. Subsequently, both Yb^3+^ ions can emit a photon with an energy of approximately 10,000 cm^−^^1^. Effectively, this process cuts a single blue photon into two infrared photons with sufficient energy to be absorbed by crystalline Si (Fig. 1d)^10–12^.

Evidence for the cooperative mechanism has been scarce to absent^12^. The near match between the energy of the Tm^3+ 1^G_4_ level and double the energy of the Yb^3+ 2^F_5/2_ level suggests the possibility of cooperative energy transfer^21–23^, but this alone is not proof. In fact, the observation of strong Yb^3+^ emission in a sample with an Yb^3+^ doping concentration as low as a few percent^21–23^ is inconsistent with cooperative energy transfer. Indeed, efficient cooperative energy transfer occurs only if a Tm^3+^ ion is in nearest-neighbour proximity to two Yb^3+^ ions in the crystal, which is unlikely unless the Yb^3+^ doping concentration exceeds ~25%^8^.

An alternative Tm^3+^-to-Yb^3+^ energy-transfer mechanism could be cross-relaxation (Fig. 1e)^12,32^: Tm^3+^ in the excited ^1^G_4_ level transfers part of its energy to a nearby Yb^3+^ ion. Tm^3+^ thereby relaxes to the intermediate ^3^H_5_ level and Yb^3+^ is excited to the ^2^F_5/2_ level. Although Yb^3+^ can subsequently emit a photon that can be absorbed by crystalline Si, the energy of the ^3^H_5_ level (8500 cm^−^^1^) is lower than the bandgap of Si. Hence, Tm^3+^-to-Yb^3+^ cross-relaxation yields at most one useful photon (Fig. 1f) for Si-based photovoltaics.

Tm^3+^-to-Yb^3+^ cross-relaxation may seem unlikely, as the energy mismatch between the Tm^3+ 1^G_4_ → ^3^H_5_ and Yb^3+ 2^F_7/2_ → ^2^F_5/2_ transitions is as large as 2000–3000 cm^−1^ (compare Fig. 1a, b)^31^. This energy mismatch could, however, be bridged by multiphonon emission. As multiphonon processes generally become less efficient as the number of phonons involved increases^28^, one may expect a strong effect of the host material on the occurrence of cross-relaxation. To test and exploit this, we investigated the Tm^3+^-to-Yb^3+^ energy transfer in a series of host materials with different phonon energies (Fig. 1g–j). Specifically, we prepared microcrystalline Tm^3+^/Yb^3+^-codoped YBO_3_ (Fig. 1a, b, g; highest phonon energy of 1050 cm^−1^)^33,34^, YAG (Fig. 1h; 860 cm^−1^)^35,36^, Y_2_O_3_ (Fig. 1i; 600 cm^−1^)^37^, and NaYF_4_ (Fig. 1j; 370 cm^−1^)^38^. In these materials, Tm^3+^-to-Yb^3+^ cross-relaxation would be a two-phonon-, three-phonon-, four-phonon-, or six-phonon-assisted process, respectively, considering a mismatch of ~2000 cm^−1^ between the closest crystal-field components of the transitions involved (see Fig. 1a). A series of samples was prepared for each material with systematically varied Yb^3+^ concentration. The Tm^3+^ concentrations were chosen to be low enough to minimize Tm^3+^-to-Tm^3+^ cross-relaxation^39,40^ but sufficiently high to obtain a sufficient luminescence signal. X-ray diffraction (XRD) analysis (Supplementary Fig. S1, Supplementary Information) confirmed the synthesis of phase-pure samples for all Yb^3+^ concentrations. The different crystallite sizes (Fig. 1g–j) in the range of 100 nm–1 μm have negligible influence on the energy-transfer interactions, because these occur mostly at distances of 1 nm and shorter. We have previously found only minor influences of the crystal size even for particles as small as 2 nm in radius^41^.

**Fig. 2:**
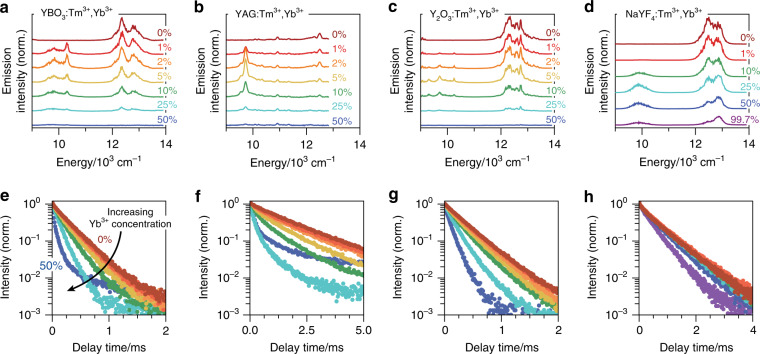
Emission spectra and decay dynamics as a function of Yb^3+^ concentration. **a** Emission spectra of YBO_3_ doped with 0.1% Tm^3+^ and increasing Yb^3+^ concentration as indicated in the plot, excited in the Tm^3+ 1^G_4_ level. The emission bands at 10,000 cm^−1^ and 12,000 cm^−1^ are due to radiative relaxation of the Yb^3+ 2^F_5/2_ and Tm^3+ 1^G_4_ levels (with overlap of ^3^H_4_ → ^3^H_6_ emission), respectively. **b** Same as in **a**, but for YAG. The Tm^3+^ concentration is 0.03% in these samples. **c** Same as in **a**, but for Y_2_O_3_. The Tm^3+^ concentration is 0.1% in these samples. **d** Same as in **a**, but for NaYF_4_. The Tm^3+^ concentration is 0.3% in these samples. **e**–**h** Photoluminescence decay curves of the ^1^G_4_ level measured for the ^1^G_4_ → ^3^F_4_ emission (~650 nm) from the same samples studied in **a**–**d**, using the same colour coding. These results show accelerated decay due to energy transfer at increasing Yb^3+^ concentration. All experiments were performed by exciting Tm^3+^ to the ^1^G_4_ level (~465 nm)

The emission spectra of the four Tm^3+^/Yb^3+^-codoped materials are qualitatively similar (Fig. 2a–d). All four materials show an emission feature centred at 12,500 cm^−1^ that is strongest at 0% Yb^3+^ (dark red) and becomes weaker for higher Yb^3+^ concentrations (from red to blue/purple). This emission originates from the ^1^G_4_ → ^3^H_5_ and ^3^H_4_ → ^3^H_6_ transitions of Tm^3+^ (compare Fig. 1a). The decreasing intensity is further confirmation of Tm^3+^-to-Yb^3+^ energy transfer, which becomes more efficient at higher Yb^3+^ concentrations. Indeed, the emission feature at ~10,000 cm^−1^, due to the Yb^3+ 2^F_5/2_ → ^2^F_7/2_ transition (compare Fig. 1a), increases in intensity with increasing Yb^3+^ concentration in all materials. However, for the highest Yb^3+^ concentrations (>10% in Fig. 2a–c or >25% in Fig. 2d), the emission at 10,000 cm^−1^ is partly quenched. We ascribe this to concentration quenching. The line shapes of Tm^3+ 1^G_4_ → ^3^H_5_, Tm^3+ 3^H_4_ → ^3^H_6_, and Yb^3+ 2^F_5/2_ → ^2^F_7/2_ are different for the four materials but consistent with previous literature reports^42,43^. The differences are due to the different crystal fields experienced by the optically active lanthanides, which split the spin–orbit levels and affect the transition energies and rates differently in each material.

Identifying the mechanism and quantifying the efficiency of Tm^3+^-to-Yb^3+^ energy transfer requires measurement of the emission decay dynamics. As expected, we observe that for all four materials, the excited-state lifetime of the Tm^3+ 1^G_4_ level decreases with increasing Yb^3+^ concentration (Fig. 2e–h). This confirms energy transfer from the Tm^3+ 1^G_4_ level to Yb^3+^. At higher Yb^3+^ concentrations, Tm^3+^ ions have (on average) more and closer Yb^3+^ neighbours, so the energy-transfer rates are higher. At the highest Yb^3+^ concentrations (50% in Fig. 2e, f), the Tm^3+^ emission intensity is strongly quenched, so the signal-to-background ratio in the photoluminescence decay measurement is poor. Comparing the measurements on the different materials, we note that the decay dynamics depend less strongly on the Yb^3+^ concentration in NaYF_4_ (Fig. 2h) than in the other materials (Fig. 2e–g). This is our first indication that the Tm^3+^-to-Yb^3+^ energy transfer depends on the maximum phonon energy of the host material.

**Fig. 3:**
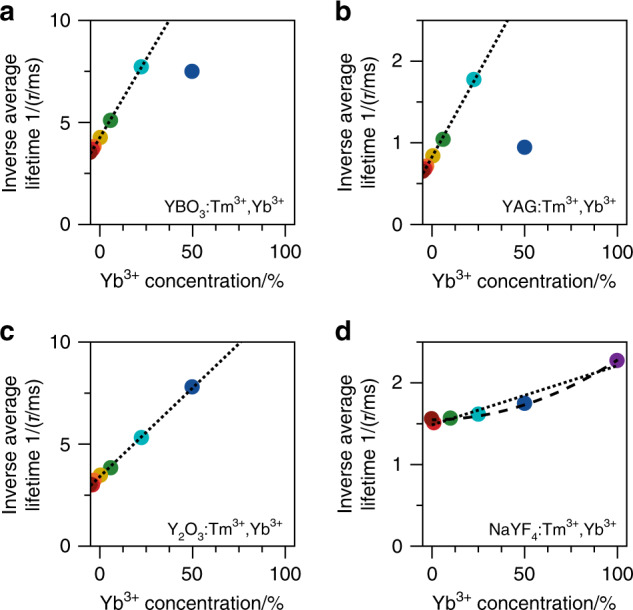
The average Tm^3+^-to-Yb^3+^ energy transfer rate. Inverse of the average lifetime of the Tm^3+ 1^G_4_ level in (**a**) YBO_3_, (**b**) YAG, (**c**) Y_2_O_3_, and (**d**) NaYF_4_ as a function of Yb^3+^ concentration. These values are extracted from the photoluminescence decay curves shown in Fig. 2e–h. The dotted lines are linear fits, and the dashed line in panel **d** is a quadratic fit

As a first analysis of the energy-transfer mechanisms in the four materials, we use the data of Fig. 2e–h and evaluate the average lifetime of the Tm^3+ 1^G_4_ level, $\langle \tau \rangle = \sum\nolimits_i {{I_i}{t_i}/} \sum\nolimits_i {{I_i}}$, where *I*_*i*_ is the emission intensity at delay time *t*_*i*_ and the summation runs over all data points *i* constituting the photoluminescence decay curve as a function of Yb^3+^ concentration. The inverse of the average lifetime (Fig. 3a–d) is approximately equal to the average decay rate of the ^1^G_4_ level,

1 $$\left\langle \tau \right\rangle ^{ - 1} \,\approx k_0 + \left\langle {k_{{\mathrm{ET}}}} \right\rangle$$

where the first term *k*_0_ is due to relaxation processes *not* involving Yb^3+^, e.g., radiative decay or multiphonon relaxation, and the second term *k*_ET_ is due to energy transfer to Yb^3+^. The *k*_ET_ of a Tm^3+^ ion depends on the number of Yb^3+^ neighbours and hence on the Yb^3+^ doping concentration *x* in the crystal. Indeed, for all four host materials, *τ*^−1^ shows a constant offset *k*_0_ and a second term *k*_ET_ that increases with Yb^3+^ concentration. The outlying data points for the highest Yb^3+^ concentration in YBO_3_ and YAG (Fig. 3a, b) are due to the low signal-to-background ratio for these measurements, which makes accurate calculation of *τ* difficult.

The results of Fig. 3a–d indicate a qualitative difference in the energy-transfer mechanism between the higher-phonon-energy hosts—YBO_3_, YAG, and Y_2_O_3_—and the lowest-phonon-energy host NaYF_4_. In the higher-phonon-energy hosts, *k*_ET_ scales linearly with Yb^3+^ concentration *x* (dotted lines in Fig. 3a–c). This indicates the occurrence of cross-relaxation (Fig. 1e, f), which is a first-order process that scales linearly with the acceptor concentration. In contrast, NaYF_4_ shows a quadratic trend (dashed line in Fig. 3d). More precisely, fitting the power of the $k_{{\mathrm{ET}}} \propto x^p$ relationship (not shown) yields *p* = 1.8, close to a value of 2. This is consistent with cooperative energy transfer (Fig. 1c, d), which is a second-order process.

For further confirmation and quantification of the energy-transfer process, we turn to Monte Carlo modelling of the ^1^G_4_ decay dynamics^8,44^. As the energy-transfer rates scale strongly with the distance between the donor and acceptor and Tm^3+^ and Yb^3+^ dopants randomly substitute Y^3+^ cation sites in the host crystal, we expect that different Tm^3+^ ions in the crystal exhibit different energy-transfer rates. For a particular donor–acceptor pair, the energy-transfer rate for cross-relaxation via dipole–dipole coupling is

2 $$k_{{\mathrm{ET}}} = \frac{{C_{{\mathrm{xr}}}}}{{r^6}}$$

where *r* is the donor–acceptor separation and *C*_xr_ is a prefactor describing the overall strength of cross-relaxation. Cooperative energy transfer requires one donor and two acceptors and scales as

3 $$k_{{\mathrm{ET}}} = \frac{{C_{{\mathrm{coop}}}}}{{r_1}^6{r_2}^6}$$

where *r*_1_ is the distance from the donor to acceptor 1, *r*_2_ is the distance from the donor to acceptor 2, and *C*_coop_ is the strength of cooperative energy transfer^8^. To model the energy transfer dynamics in a Tm^3+^/Yb^3+^-codoped sample, we Monte Carlo simulate dopant configurations and calculate from these the distribution of energy-transfer rates following Eqs. (2) and (3). More details of the model can be found in the Experimental section.

**Fig. 4:**
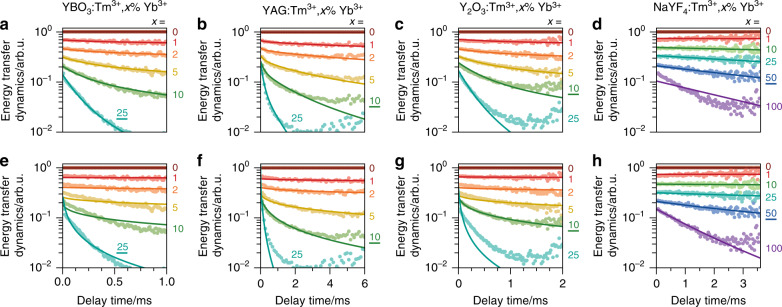
Monte Carlo modelling of energy transfer. Tm^3+^-to-Yb^3+^ energy-transfer dynamics for different Yb^3+^ concentrations in (**a**, **e**) YBO_3_, (**b**, **f**) YAG, (**c**, **g**) Y_2_O_3_, and (**d**, **h**) NaYF_4_. The energy-transfer dynamics are isolated from the full photoluminescence decay curves (Fig. 2e–h) by dividing the data obtained for *x*% Yb^3+^ by the data for 0% Yb^3+^. Panels **a**–**d** show the results of a fit to a model of phonon-assisted cross-relaxation (Eq. (2)), whereas **e**–**h** show those for a model of cooperative energy transfer (Eq. (3)). See the Experimental section for details of the modelling procedure. The phonon-assisted cross-relaxation model matches the dynamics of YBO_3_, YAG, and Y_2_O_3_, while the cooperative model matches the dynamics of NaYF_4_

Figure 4 shows the Tm^3+^-to-Yb^3+^ energy-transfer dynamics in our samples. We isolate the Tm^3+^-to-Yb^3+^ energy-transfer dynamics from the total photoluminescence decay curves (Fig. 2e–h) by following the procedure introduced in Ref. ^41^: we divide each decay curve of a sample with *x*% Yb^3+^ by the decay curve of the corresponding sample with 0% Yb^3+^. We thus use the sample with 0% Yb^3+^ as a reference to remove the dynamics due to radiative decay of Tm^3+^ and Tm^3+^-to-Tm^3+^ from our data. Solid lines are fits to the Monte Carlo model for cross-relaxation (Eq. 2; Fig. 4a–d) or to the model for cooperative energy transfer (Eq. (3) and Fig. 4e–h). For each host material, we first determine the optimal values of *C*_xr_ and *C*_coop_ for one Yb^3+^ concentration, as underlined in Fig. 4. Then, keeping the values found fixed, we plot the calculated decay curves for the other concentrations. The cross-relaxation model well matches the data for YBO_3_, YAG, and Y_2_O_3_ (Fig. 4a–c), while the cooperative model predicts too slow a decay at low Yb^3+^ concentrations and too rapid a decay at high Yb^3+^ concentrations (Fig. 4e–g). In contrast, for NaYF_4_, the cooperative model works well (Fig. 4h), whereas the cross-relaxation model shows deviations from the experimental data (Fig. 4d).

The quantitative analysis of Fig. 4 confirms that the energy-transfer mechanisms are different between the higher-phonon-energy hosts (YBO_3_, YAG, and Y_2_O_3_) and NaYF_4_. Cross-relaxation occurs in the higher-phonon-energy hosts, with rates comparable to radiative decay at Yb^3+^ concentrations as low as a few percent. In contrast, in NaYF_4_, the Tm^3+^-to-Yb^3+^ energy transfer is weak until high Yb^3+^ concentrations of >25%, and cooperative energy transfer dominates over radiative decay only at higher concentrations. Hence, we must conclude that NaYF_4_ allows for cooperative Tm^3+^-to-Yb^3+^ energy transfer not because the rate of this process is particularly high but rather because cross-relaxation is strongly suppressed.

## Discussion

**Fig. 5:**
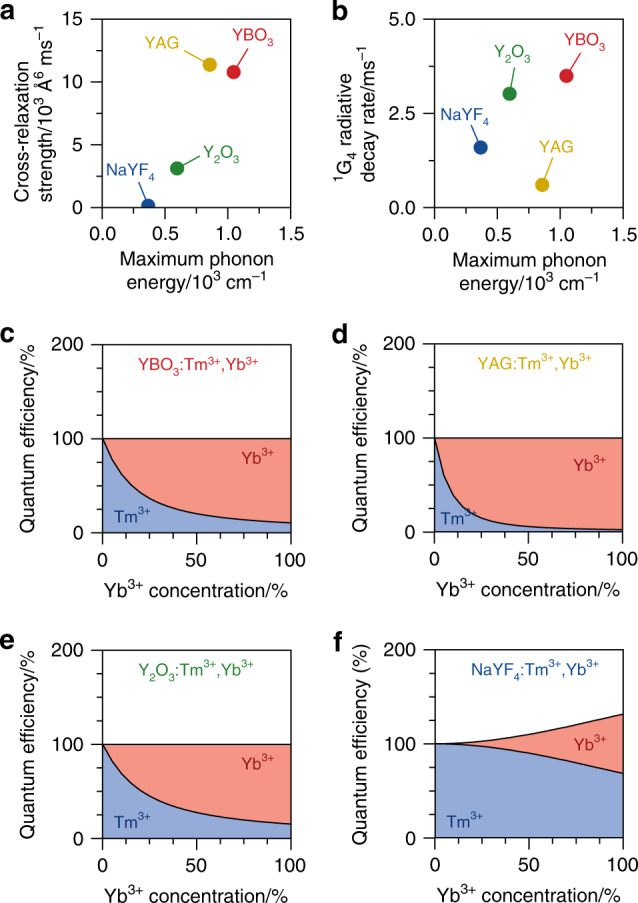
Comparing the photon-cutting potential of different host materials. **a** Fitted Tm^3+^-to-Yb^3+^ cross-relaxation strength *C*_xr_ (see Eq. (2)) for the different host materials, plotted as a function of the maximum phonon energy of the host. **b** Corresponding intrinsic decay rates of the ^1^G_4_ level. **c** Calculated maximum quantum efficiency of YBO_3_:Tm^3+^,Yb^3+^ as a function of Yb^3+^ concentration based on our phonon-assisted cross-relaxation model. We count only emission that can be absorbed by crystalline Si, i.e., visible emission from the Tm^3+ 1^G_4_ level (blue-shaded area) and near-infrared emission from Yb^3+^ (red-shaded area), and assume zero nonradiative decay from the Yb^3+ 2^F_5/2_ level. **d** Same, but for YAG:Tm^3+^,Yb^3+^. **e** Same, but for Y_2_O_3_:Tm^3+^,Yb^3+^. **f** Same, but for NaYF_4_:Tm^3+^,Yb^3+^ and using our model for cooperative energy transfer. Of the four materials studied, only NaYF_4_:Tm^3+^,Yb^3+^ is a photon-cutting phosphor that can produce two near-infrared photons (useful for crystalline Si) from a single blue photon absorption event

We can determine how weak the cross-relaxation is in NaYF_4_ compared to the other hosts by analysing the measurements at low Yb^3+^ concentrations (≤25%) using the cross-relaxation model. This yields values for the Tm^3+^-to-Yb^3+^ cross-relaxation strength in NaYF_4_ of *C*_xr_ = (7 ± 6) × 10^1^ Å^6^ ms^−1^. Cross-relaxation in NaYF_4_ is thus two orders of magnitude slower than that in the higher-phonon-energy hosts (Fig. 5a). This is consistent with the large energy mismatch between the ^1^G_4_ → ^3^H_5_ and ^2^F_7/2_ → ^2^F_5/2_ transitions involved in cross-relaxation (compare Fig. 1a, b). Our experiments show that the 2000–3000 cm^−^^1^ mismatch can be bridged by a two-, three-, or four-phonon process in YBO_3_, YAG, or Y_2_O_3_, respectively. In contrast, the six-phonon-assisted cross-relaxation in NaYF_4_ is too slow to compete with other decay pathways from the Tm^3+ 1^G_4_ level. Closer inspection of Fig. 5a reveals that four-phonon-assisted cross-relaxation in Y_2_O_3_ is already slower by a factor of 3 than the corresponding lower-order processes in YBO_3_ and YAG. Qualitatively, such a strong dependence of the cross-relaxation rates on the number of phonons involved is expected from the exponential energy-gap law for nonradiative relaxation^28^. Quantitatively, however, the relation between the number of phonons involved and the cross-relaxation strength (*C*_xr_) is not straightforward, as *C*_xr_ depends on various other factors such as the transition dipole moments of the electronic and vibrational transitions involved^28,45^. In general, lanthanide f–f transition dipole moments are different for different host materials, as they are strongly dependent on the crystal-field symmetry and covalency of the host material^45^. This explains why the intrinsic ^1^G_4_ decay rates *k*_0_ are different for the different host materials (Fig. 5b) and why *C*_xr_ does not monotonically increase with phonon energy (Fig. 5a). Future work may reveal that temperature further affects the delicate competition between phonon-assisted cross-relaxation and cooperative energy transfer.

The energy-transfer mechanism, the corresponding energy-transfer strength (*C*_xr_ or *C*_coop_; Eqs. (2) and (3)), and the decay rate *k*_0_ of the ^1^G_4_ level at 0% Yb^3+^ (Fig. 5b) determine the maximum quantum efficiency of visible-to-near-infrared photon-conversion achievable with a particular host material. In our definition of quantum efficiency, we include only the emission of photons that can be absorbed by crystalline Si. Creating two of these photons from a single Tm^3+^ ion in the ^1^G_4_ level requires cooperative energy transfer rather than cross-relaxation. To calculate the maximum quantum efficiency, we first construct the theoretical normalised photoluminescence decay curve of the ^1^G_4_ level for each host material for any arbitrary Yb^3+^ concentration:

$$I\left( t \right) = {\mathrm{e}}^{ - k_0t}T\left( t \right)$$

where *T*(*t*) is the multiexponential decay function due to energy transfer (see the ‘Methods’ section for details). The theoretical quantum efficiency is then given by

$$\eta = \eta _{{\mathrm{Tm}}} + \eta _{{\mathrm{Yb}}}$$

Herein,

$$\eta _{{\mathrm{Tm}}} = k_0{\int \nolimits_0^\infty} I\left( t \right){\mathrm{d}}t$$

is the efficiency of Tm^3+ 1^G_4_ emission, and

$$\eta _{{\mathrm{Yb}}} = Q\left( {1 - \eta _{{\mathrm{Tm}}}} \right)$$

is the efficiency of Yb^3+^ emission. The factor *Q* depends on the dominant energy-transfer mechanism in the host material. Its value is *Q* = 1 for the cross-relaxation process in YBO_3_, YAG, and Y_2_O_3_ or *Q* = 2 for cooperative energy transfer in NaYF_4_.

In Fig. 5c–f, we plot the maximum quantum efficiency for the different host materials as a function of Yb^3+^ concentration, calculated with our Monte Carlo model. We neglect intrinsic losses in Tm^3+^ due to nonradiative decay or infrared emissions as well as concentration quenching effects of the Yb^3+^ emission (see Fig. 2a–d). The calculations of Fig. 5c–f thus show the highest possible quantum efficiency that could be achieved if the materials are optimized to suppress any loss channel. As expected, the Yb^3+^ emission rapidly increases with increasing Yb^3+^ concentration in the higher-phonon-energy hosts (Fig. 5c–e), but the overall quantum efficiency never exceeds unity. In contrast, in NaYF_4_, the Yb^3+^ emission increases more slowly but pushes the overall quantum efficiency up to 132% in NaYbF_4_:Tm^3+^.

Our findings highlight the possibility of qualitatively altering the energy-conversion pathways in lanthanide-doped crystals by choosing a host material with the appropriate phonon spectrum. This allows us to change the blue-to-near-infrared conversion by the Tm^3+^/Yb^3+^ couple from a simple downshifting process in the higher-phonon-energy host materials into a photon-cutting process in the lower-phonon-energy host NaYF_4_. Only photon cutting in the NaYF_4_ host holds promise for enhancement of the current output of crystalline Si solar cells because it can convert blue photons into near-infrared photons of ~1000 nm with a quantum efficiency exceeding unity. Similar qualitative differences between host materials may be expected in terms of the (often very complicated) pathways of photon upconversion^46^. While previous studies have claimed achievement of high photon-cutting efficiencies with the Tm^3+^/Yb^3+^ couple in a wide variety of host materials, our findings show that photon cutting is only possible in host lattices with phonon energies not exceeding ~400 cm^−1^.

## Materials and methods

### Chemicals and materials

All chemicals were used without further purification. Y_2_O_3_ (99.999%) was purchased from Alfa Aesar; Tm_2_O_3_ (99.999%) from Heraeus; Yb_2_O_3_ (99.99%), Y_2_O_3_ (99.99%), Al(NO_3_)_3_.9H_2_O (≥98%), urea (BioReagent), and nitric acid (HNO_3_; puriss. p.a., ≥65%) from Sigma-Aldrich; and boric acid (H_3_BO_3_; ≥99.5%) from Merck.

### Synthesis of β-NaYF_4_:Tm^3+^,Yb^3+^ microcrystalline phosphors

*β*-NaYF_4_:0.3%Tm^3+^,*x*%Yb^3+^ powder samples were synthesized following the approach developed by Krämer et al.^4^.

### Combustion synthesis of Y_2_O_3_-, YAG-, and YBO_3_-based polycrystalline phosphors

A urea–nitrate solution combustion process was used for the synthesis of a series of polycrystalline powder phosphors of Y_2_O_3_, YAG, and YBO_3_ codoped with Tm^3+^ and Yb^3+^. Y_2_O_3_, Tm_2_O_3_, and Yb_2_O_3_ were used as lanthanide (Ln) sources, Al(NO_3_)_3_.9H_2_O as the Al source for YAG, H_3_BO_3_ as the B source for YBO_3_, and urea as the organic fuel for the combustion reaction. Stoichiometric amounts of Y_2_O_3_, Tm_2_O_3_, and Yb_2_O_3_ were dissolved in nitric acid to obtain aqueous solutions of mixed Ln(NO_3_)_3_. For the synthesis of Y_2_O_3_:Tm^3+^,Yb^3+^, solid urea (molar ratio urea/Ln = 2:1) was added to the Ln(NO_3_)_3_ solution. For YAG:Tm^3+^,Yb^3+^, an Al(NO_3_)_3_ solution (molar ratio Al/Ln = 5:3) and urea (molar ratio urea/Ln = 5:1) were added to the Ln(NO_3_)_3_ solution. For YBO_3_:Tm^3+^,Yb^3+^, solid urea (molar ratio urea/Ln = 3:1) and H_3_BO_3_ (5% molar excess) were added to the Ln(NO_3_)_3_ solution. After vigorous stirring for 20 min at approximately 70 °C, the resulting homogeneous precursor solution was placed in a preheated furnace at 500 °C in air to initiate the combustion reaction. Amorphous solid precursors formed from the solutions within a few minutes. Finally, annealing in ambient atmosphere at 1000 °C for 4 h, 1500 °C for 10 h, and 900 °C for 4 h produced crystalline Tm^3+^/Yb^3+^-codoped Y_2_O_3_, YAG, and YBO_3_, respectively.

### Characterization

Phase identification of all the prepared products was performed on a Philips PW1700 X-ray powder diffractometer using Cu K-α (*λ* = 1.5418 Å) radiation. XRD patterns were collected over a 2*θ* range from 10° to 80° at an interval of 0.02°. The morphology of the samples was checked using high-resolution scanning electron microscopy (Phenom ProX Desktop SEM, 10 keV) and a thin layer of sample powder on conducting carbon tape. Steady-state emission and excitation spectra were recorded using an Edinburgh Instruments FLS920 spectrophotometer equipped with different excitation sources, including a 450 W xenon lamp and an optical parametric oscillator laser (OPO; Opolette HE 355II; 20 Hz; pulse width ~7 ns), TMS300 monochromators, a thermoelectrically cooled R928 photomultiplier tube (PMT) for visible wavelengths, and a liquid-nitrogen-cooled R5509-72 PMT for near-infrared wavelengths. Photoluminescence decay curves were measured using multichannel scaling on the Edinburgh Instruments FLS920 spectrophotometer under pulsed OPO laser excitation.

### Modelling the energy-transfer dynamics

We used the Monte Carlo procedure to model the dynamics of the cross-relaxation and cooperative energy transfer described in detail in Ref. ^44^. Briefly, for each host material, we randomly generated several thousand different environments of an excited Tm^3+^ ion, i.e., a number of nearest Yb^3+^ neighbours, next-nearest neighbours, etc., taking into account the overall Yb^3+^ doping concentration. NaYF_4_ and Y_2_O_3_ have two possible crystal sites for the central Tm^3+^ ion, which were weighted by the relative occurrence. We made the simplification that the energy-transfer strengths *C*_xr_ and *C*_coop_ are the same for all sites in the crystal structures. Next, for each environment *i*, we calculated the total energy-transfer rate *k*_ET,*i*_ by summing over all (pairs of) acceptors (see Eqs. (2) and (3)) and obtained an expression $T\left( t \right) = A\mathop {\sum}\nolimits_i {e^{ - k_{{\mathrm{ET}},i}t}}$ for the multiexponential energy-transfer dynamics. We determined the best value for *C*_xr_ (Eq. (2)) or *C*_coop_ (Eq. (3)) by fitting our model to the data for one of the Yb^3+^ concentrations, indicated in Fig. 4 by the underlined value for *x*. Finally, we fitted our model to the data for all other Yb^3+^ concentrations, only optimizing the amplitudes *A* while keeping *C*_xr_ or *C*_coop_ fixed.

## Supplementary information

Supplementary Information
