# Supplementary material for: Understanding and tuning blue-to-near-infrared photon cutting by the Tm3+/Yb3+ couple
Source: Light Sci Appl. 2020 Jun 19;9:107. doi: 10.1038/s41377-020-00346-z (PMC7305182; doi:10.1038/s41377-020-00346-z)
Supplement: Supplementary file 1 — Supplementary Information [file 41377_2020_346_MOESM1_ESM.pdf]

# Supplementary Information

# Understanding and tuning blue-to-near-infrared photon cutting by the $Tm^{3+}/Yb^{3+}$ couple

Dechao Yu,[1,†] Ting Yu,[1,2,†] Arnoldus J. van Bunningen,[1] Qinyuan Zhang,[2,*] Andries Meijerink,[1] and Freddy T. Rabouw[1,*]

[1]*Debye Institute for Nanomaterials Science, Utrecht University, Princetonplein 1, 3584 CC Utrecht, The Netherlands*

[2]*State Key Laboratory of Luminescence Materials and Devices, and Institute of Optical Communication Materials, South China University of Technology, Guangzhou 510641, China*

[†]These authors contributed equally to this work.

*Author to whom correspondence should be addressed; electronic mail:

qyzhang@scut.edu.cn and f.t.rabouw@uu.nl

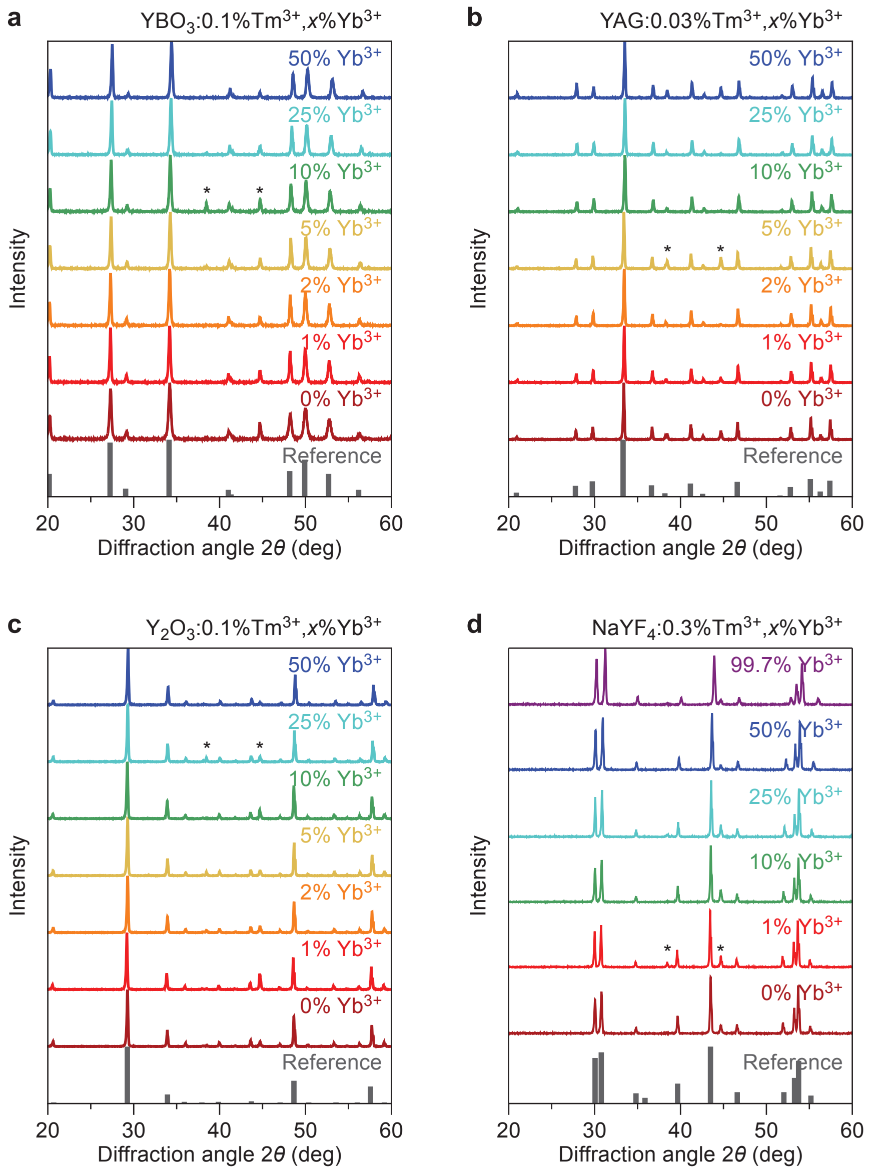


**Figure S1:** X-ray diffraction (XRD) patterns of our **(a)** $YBO_3$:0.1%$Tm^{3+}$,*x*%$Yb^{3+}$, **(b)** YAG:0.03%$Tm^{3+}$,*x*%$Yb^{3+}$, **(c)** $Y_2O_3$:0.1%$Tm^{3+}$,*x*%$Yb^{3+}$, and **(d)** $NaYF_4$:0.3%$Tm^{3+}$,*x*%$Yb^{3+}$ samples, recorded with a Cu K-α X-ray source. The diffraction peaks at 39 and 45 degrees (indicated with asterisks in each of the panels) originate from the Al sample holder. Gray bars indicate the reference patterns PDF cards no. 16-0277, 033-0040, 01-0831, and 16-0334, respectively.
